# Genotoxic evaluation of chemical fractions of *Cymbopogon citratus* (DC) Stapf using plasmid assay

**DOI:** 10.3389/fphar.2026.1897115

**Published:** 2026-10-02

**Authors:** Mayda Carrasco-Alfonso, Fabiana Fuentes-León, Laura Sánchez-Hechavarría, Claudia Rodríguez-Hernández, Ángel Sánchez-Lamar, Antonio Francioso

**Affiliations:** 1 Plant Biology Department, Faculty of Biology, University of Havana, La Habana, Cuba; 2 Dirección de Oncología Experimental y Toxicología, Centro de Investigación y Desarrollo de Medicamentos (CIDEM), La Habana, Cuba; 3 Department of Bioscience and Technology for Food, Agriculture and Environment, University of Teramo, Teramo, Italy; 4 Department of Bioorganic Chemistry, Leibniz Institute of Plant Biochemistry (IPB), Halle (Saale), Germany; 5 Instituto Universitario de Bio-Orgánica Antonio González, University of La Laguna, San Cristóbal de La Laguna, Tenerife, Spain; 6 Institute of Biological Chemistry, Sapienza University of Rome, Rome, Italy

**Keywords:** chemical fractions, DNA strand breaks, natural products, oxidative damage, phytocomponents *Cymbopogon citratus*, phytochemistry

## Abstract

**Introduction:**

*Cymbopogon citratus* (DC.) Stapf is a medicinal plant widely used in traditional medicine in Africa, Asia and Latin America, mainly consumed as aqueous decoctions or infusions to treat fever, colds, cough, gastrointestinal disorders, hypertension, inflammation and anxiety. Despite its extensive traditional use, information on the genotoxic and oxidative effects of its individual chemical fractions remains limited.

**Methods:**

The DNA strand breaks and oxidative effects of the aqueous, butanolic and essential oil fractions obtained from the aqueous extract of *C. citratus* were evaluated using an ex vivo purified pCMUT plasmid DNA model. Cleavage was analyzed after short- and long-term exposure, and oxidative damage was assessed using formamidopyrimidine-DNA glycosylase (FPG). Phytochemical characterization of the aqueous and butanolic fractions was performed by HPLC–HR-MS/MS.

**Results:**

None of the fractions induced plasmid cleavage after 30 min of exposure. However, the aqueous fraction caused significant DNA strand breaks after prolonged incubation. Oxidative DNA damage was detected with the aqueous and essential oil fractions, whereas the butanolic fraction showed no detectable oxidative or breaking effects. Phytochemical analysis revealed qualitative and quantitative differences between fractions, particularly in phenolic acids and flavonoids.

**Discussion:**

The aqueous fraction of *C. citratus* exhibits a higher genotoxic and pro-oxidant potential under prolonged exposure, while the butanolic fraction presents a safer profile under the evaluated conditions. These findings highlight the importance of fraction-specific toxicological assessment to support the safe traditional and pharmacological use of *C. citratus*.

## Graphical Abstract

**Figure:**
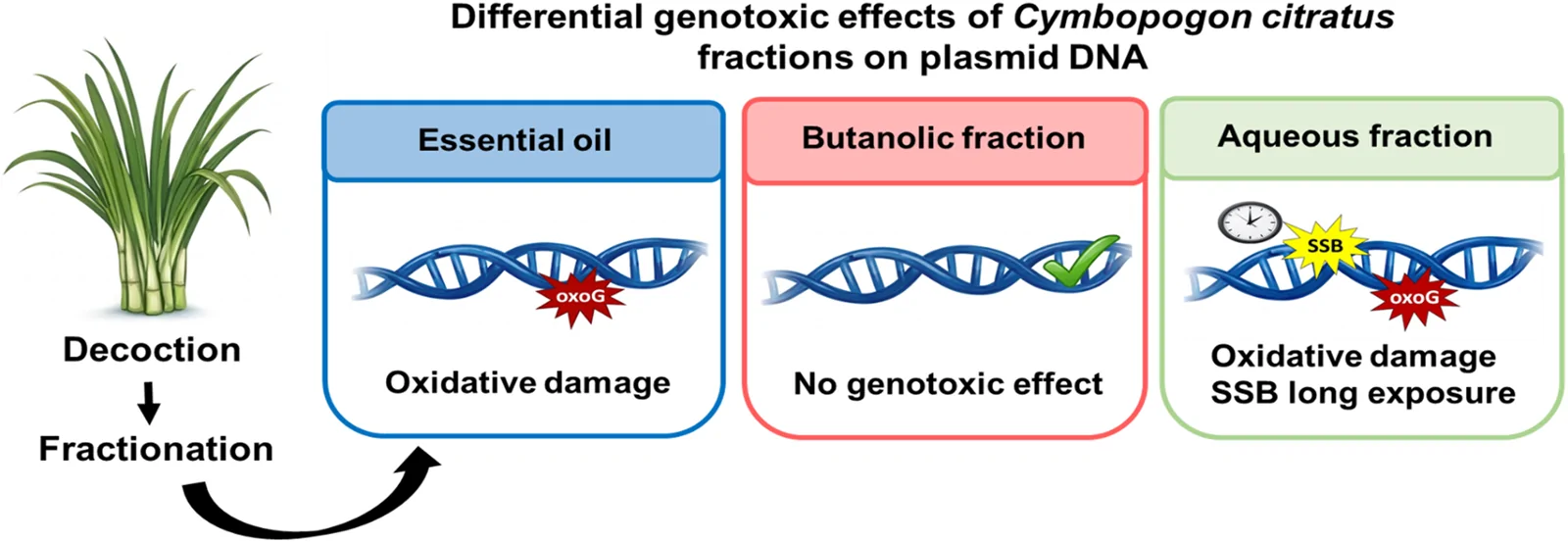

## 1 Introduction

Since ancient times, humans have turned to the use of plants for therapeutic purposes, making this practice a fundamental component of the earliest forms of healthcare. Although it initially lacked systematic scientific validation, the tangible benefits of natural medicine have persisted throughout history. In the 19th century, the active chemical components responsible for the therapeutic effects of numerous plant species were discovered and isolated, ushering in a revolution in the identification of novel bioactive compounds from natural sources. This advancement fostered the development of new pharmacological research approaches focused on secondary plant metabolites such as polyphenols, terpenes, and flavones, among others. Today, plants remain an indispensable source of biologically active molecules used in the formulation of medicaments, nutritional supplements, and natural cosmetics (Chaachouay and Zidane, 2024).

Despite their natural origin, medicinal plants face several challenges. While they often act on multiple physiological systems and are generally considered safe and non-toxic, natural compounds are not inherently risk-free (Chaachouay and Zidane, 2024). Therefore, toxicity studies are essential before justifying their pharmaceutical or cosmeceutical application.

*Cymbopogon citratus* (DC) Stapf, commonly known in Cuba as “Caña Santa”, is one of the most widely consumed medicinal plants worldwide, typically prepared as a decoction due to its pleasant flavor and its well-established healing properties (Brull and Cedeño, 2026, Menéndez-Perdomo et al., 2018). In traditional medicine, decoctions or infusions of *C. citratus* leaves are widely used in Africa, Asia and Latin America for the treatment of fever, colds and cough, gastrointestinal spasms and stomach ache, hypertension, rheumatism and anxiety, among other conditions. Several scientific studies have confirmed that *C. citratus* exhibits analgesic, anti-inflammatory, antioxidant, antihypertensive, antifungal, anticancer, antinociceptive, and anxiolytic properties. *Cymbopogon citratus* has also demonstrated antimutagenic activity, particularly radioprotective against gamma radiation and ultraviolet-C (UVC) light reviewed in (Menéndez-Perdomo et al., 2018). The photoprotective properties of aqueous *C. citratus* extract have been evaluated using an *ex vivo* plasmid DNA assay (González-Pumariega et al., 2016) and *in vitro via* the SOS Chromotest in *Escherichia coli* (*E. coli*) (Vernhes et al., 2016). Phytochemical analyses of this species have identified a broad spectrum of secondary metabolites and bioactive compounds, including phenolic acids, flavonoids, stilbenes, tannins, alkaloids, as well as proteins, lipids, and sugars (Oladeji et al., 2019; Kiełtyka-Dadasiewicz et al., 2024).

Chemical bioassay-guided fractionation is the laboratory strategy that enables the study of phytocomponents that may otherwise volatilize or be present at insufficient concentrations in the total plant extract. This procedure allows for the evaluation of less complex fractions, thereby facilitating the identification of the compounds responsible for the biological activities previously attributed to the whole extract (Chaachouay and Zidane, 2024). In this context, the total extract of *C. citratus* has been fractionated with the aim of elucidating which specific compounds contribute to its therapeutic effects. The volatile phytocomponents of *C. citratus*, including steroids and terpenoids, are primarily concentrated in the essential oil fraction(Oladeji et al., 2019; Luang-In et al., 2024; Kiełtyka-Dadasiewicz et al., 2024). Therefore, it is necessary to study the behavior of the obtained fractions as they may exhibit distinct biological activity profiles compared to the total extract, particularly in terms of toxicity.

The toxic capacities of the aqueous, butanolic, and essential oils fractions of *C. citratus*—measured in terms of cytotoxicity and genotoxicity, as primary steps in non-clinical and DNA protective studies—have been previously evaluated using bacterial models such as *E. coli* (Fuentes-León et al., 2017) and *Caulobacter crescentus* (Fuentes-León et al., 2017). However, there is a lack of information about the direct interaction with DNA molecule and the oxidative capacity. *Ex vivo* DNA-based studies allow for direct and specific evaluation of the interaction between the fractions and genetic material, eliminating the influence of complex cellular processes such as metabolism, detoxification, DNA repair, and stress-response mechanisms present in bacterial models.

This article aims to: (i) use naked plasmid DNA to evaluate the genotoxicity—understood as the ability to induce DNA strand breaks and oxidative damage—caused by chemical fractions of *C. citratus*; and (ii) perform phytochemical profiling to relate the presence of specific compounds with the observed effects. This complements obtained findings and provides a clearer understanding of the previous results.

## 2 Materials and methods

### 2.1 Collection of plant material

The leaves of *C. citratus* (DC) Stapf were collected from adult plants grown in Boyeros, Havana, Cuba. The specimens were examined and taxonomically verified at the Medicinal Plants Station in Güira de Melena, Artemisa, Cuba (voucher No. 4593).

### 2.2 Chemical fractionation of the total aqueous extract of *C. citratus*

The aqueous, butanolic and essential oil fractions were obtained by specialists at the Central Laboratory of Pharmacology of the Dr. Salvador Allende Hospital. The leaves were subjected to the decoction process for 30 min (40 g of dried leaves in 275 mL), and then water vapor entrainment fractionation was used to separate the essential oils(Elhassan et al., 2016) and the aqueous extract was subsequently partitioned with n-butanol to yield the butanolic and aqueous fractions. Each liquid fraction (aqueous and butanolic) was evaporated to remove solvents and obtain the dried extracts, which were stored in glass bulbs protected from light at 4 °C until use.

For the chromatographic and biological assays, stock solutions (16 mg/mL) were prepared from the dried extracts. The butanolic, essential oils and aqueous fractions were solubilized with 2.8%, 2% and 0% dimethyl sulfoxide (DMSO), and Mili-Q water, respectively. From these stock solutions, serial dilutions were prepared at the time of the experiment for the evaluation of 0.1, 0.5, 1.0, 2.0, and 4.0 mg/mL. These concentrations were selected to ensure consistency with previous studies and to allow the evaluation of direct interactions with DNA under controlled *ex vivo* conditions.

### 2.3 Experimental model

For *ex vivo* assays, the DNA used was the plasmid pCMUT (1762 bp), which contains bacterial replication origin, an ampicillin resistance gene, and an EcoR1 restriction site. Plasmid DNA purification was performed using the standardized QIAGEN® Plasmid Maxi Kit procedure. The purity of the obtained DNA (A_260_/A_280_ = 1.82; 81% supercoiled plasmid DNA) was corroborated by qualitative electrophoresis, comparing with standard DNA of the forms supercoiled (SF) and relaxed (RF). Quantification of the purified DNA was performed using the Thermo Scientific™ NanoDrop™ Spectrophotometer. The plasmid was used at a concentration of 100 ng/μL.

### 2.4 Evaluation of DNA strand breaks

The DNA cleavage assay followed the protocol of Vernhes et al. (2013). To 2.0 μL of DNA, was added 2.0 μL of the *C. citratus* chemical fraction being evaluated, prepared at twice the final test concentration: 0.1, 0.5, 1.0, 2.0, and 4.0 mg/mL. As a negative control, 2.0 μL of the plasmid was used, and all samples were completed to a final volume of 20 μL with Milli-Q water. A 2.0 μL sample of the plasmid previously irradiated with 300 J/m^2^ UVC was used as a positive control, to which was added 2.0 μL of T4 endonuclease V enzyme and 16 μL of NET solution and incubated at 37 °C for 1 h. Treated samples were incubated at 37 °C for 30 min prior to electrophoresis.

The effect of extended exposure time at the highest non-genotoxic concentration previously determined (4.0 mg/mL), was also evaluated. Samples were incubated for 0.5, 1, 2, and 4 h and analyzed by gel electrophoresis.

### 2.5 Assessment of oxidative damage

Prior to evaluating oxidative damage, it was necessary to assess whether the fractions inhibited the activity of formamidopyrimidine-DNA glycosylase (FPG). This enzyme recognizes and removes oxidized nitrogenous bases, particularly modified purines such as 8-oxoguanine, generating abasic sites that are subsequently converted into single-strand breaks detectable by electrophoresis.

For these assays, was utilized FPG obtained from New England Biolabs, Ipswich, MA, as described by Schuch et al., 2009. The enzyme was prepared by combining 0.1 µL of FPG, 0.2 µL of BSA reagent, and 2.0 µL of Buffer for each treatment.

Initially, 2.0 μL of the FPG enzyme were mixed with 2.0 μL of each chemical fraction of *C. citratus*, at a final concentration of 4.0 mg/mL. These treatments were incubated for 30 min at 37 °C. Subsequently, 2.0 μL of the same plasmid DNA -previously irradiated with ultraviolet-A (UVA, 500 kJ/m^2^)- was added and incubated for 1 h at 37 °C. As controls, 2.0 μL of the plasmid, UVA irradiated, was used as a negative control, and 2.0 μL of the plasmid UVA irradiated with 2.0 μL of the FPG enzyme, was used as a positive control. All samples were adjusted to a volume of 20 µL with Milli-Q water and incubated at 37 °C for 1 h before conducting electrophoresis.

Based on the previous assay results, the evaluation of oxidative damage was carried out using the same enzyme mixtures and control conditions. Each sample included 2.0 μL of non-irradiated plasmid DNA, 2.0 μL of the *C. citratus* fraction (yielding 4.0 mg/mL), and was incubated for 30 min at 37 °C. Then, 2.0 μL of FPG mixture was added, and the volume adjusted to 20 μL with Milli-Q water. Samples were incubated for 1 h at 37 °C prior to electrophoretic analysis.

### 2.6 Quantification of damage

The criterion for damage was the appearance of the RF of the plasmid in the agarose gel electrophoresis as a qualitative analysis (Schuch et al., 2009). To separate the SF and RF of plasmids, 0.8% agarose gel electrophoresis dissolved in TBE (0.5 X) with ethidium bromide (0.1 mg/μL) was developed at 100 V for 40 min in an electrophoresis chamber (Model MINIS-150VS, 2A).

Image analysis was performed using the software ImageJ, developed at the U.S. National Institute of Health (http://imagej.nih.gov/ij). In this program, the picture was processed for quantifying the intensity peaks of the bands in the gel. By calculating the area under each peak, you can obtain quantitative data that can be used for further analysis and comparison between samples.

The formula used to perform the quantitative analysis was:

$$X=-\ln\left[\left(1.4\times \text{SF})/1.4\times \text{SF}+\text{RF}\right)\right]/1.8$$

where X is the number of break sites per kbp; SF and RF are the fluorescence intensities of the supercoiled and relaxed bands; 1.4 corrects for the higher ethidium-bromide fluorescence of RF relative to SF; and 1.8 is the size-correction factor for pCMUT (kbp). 

### 2.7 Chemical analysis of the aqueous and butanolic fractions by HPLC

For the chromatographic analysis, 3.0 mg of each dried extract (Aqueous and Butanolic fraction) were dissolved in 1 mL of LC-MS grade MeOH and 2.0 µL were analyzed. LC-HR-MS/MS (High-Resolution Tandem Mass Spectrometry) measurements were performed on a Dionex Ultimate 3000 UHPLC System, equipped with a quaternary pump, autosampler (100 µL sample loop, partial injection mode, 2.0 µL injection volume, sample temperature 8 °C), a photodiode array detector (PDA) (Thermo Fisher Scientific, Bremen, Germany). Chromatographic analyses were performed on a Waters C18 HSST3 column (100 mm × 1 mm i. d., 1.7 μm particle size).

Solvent A was 0.1% aqueous HCO_2_H and solvent B was 0.1% HCO_2_H in CH_3_CN. The flow rate was 0.5 mL/min and the column temperature was set at 25 °C. Elution was performed isocratically for the first minute with 2.0% B; from min 1 to min 6 solvent B was linearly increased to 55%; from 6 to 10 min 20% A and 80% B; then, in 0.5 min solvent B was set at 100% and maintained for 2 min. The column was re-equilibrated with 98% A and 2% B before the next injection. An aliquot of the extract dried under vacuum was resuspended in 100 μL and 2.0 μL injected through the needle. The effluent from PDA detector was connected online to an LTQ-Orbitrap Elite mass spectrometer equipped with a high-temperature electrospray ionization (HESI) ion source, controlled by the Excalibur 2.7 software (Thermo Fisher Scientific, Bremen, Germany) and operated in the negative or positive ion mode.

The ion spray voltage was set to 4.0 kV, sheath and auxiliary gases on 20 and 5 psi, respectively. The Orbitrap-MS spectra were acquired at the m/z range of 50 - 2000 with a resolution of 30,000. The tandem mass spectra were acquired by collision-induced dissociation (CID) in a linear ion trap (LIT) at 35% normalized collision energy and isolation width of 2.0 m/z. The ions were detected at the FT-resolution of 30,000.

### 2.8 Statistical analysis

All experimental procedures described above were performed in triplicate using independent replicates to ensure reproducibility and statistical reliability and provided the statistical robustness required for the interpretation. Statistical analyses were conducted using STATISTICA 10 software. Data were first tested for normality using the Kolmogorov–Smirnov test and for homoscedasticity using Bartlett’s test, as premises for ANOVA analysis. To compare experimental means with their respective controls, Dunnett’s test was applied as a *post hoc* procedure. A p-value<0.05 was considered statistically significant.

## 3 Results

The genotoxicity of the chemical fractions of *C. citratus* was evaluated considering two main aspects: DNA strand breaks and potential oxidative damage.

### 3.1 Evaluation of DNA strand breaks

To assess genotoxic potential, plasmid DNA was exposed to increasing concentrations (0.1, 0.5, 1.0, 2.0, and 4.0 mg/mL) of the aqueous, butanolic, and essential oil fractions. None of the analyzed fractions induced DNA strand breaks after 30 min of incubation at 37 °C. Quantification of RF plasmids in the samples treated with the fractions did not show a significant increase compared to the negative control (C−) (Figure 1). These results were confirmed by Dunnett’s test, which showed no statistically significant differences (p > 0.05). The error bars in the graphs represent the standard error of the mean (SEM).

**FIGURE 1:**
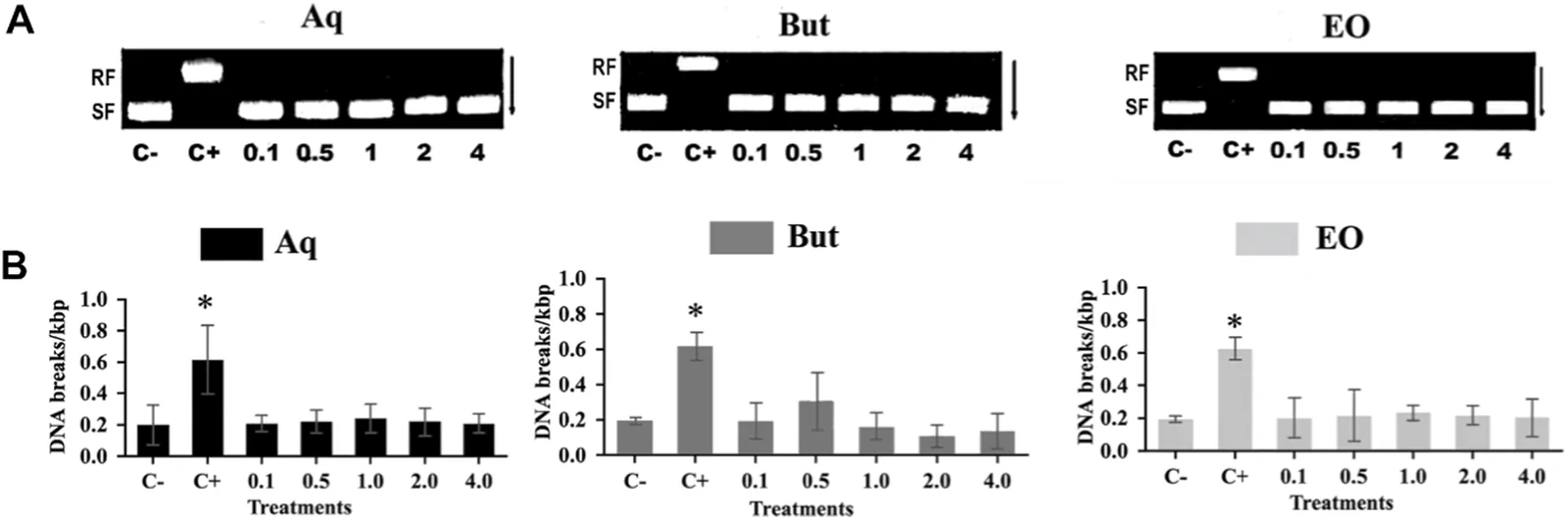
Effect of increasing concentrations of the aqueous (Aq), butanolic (But), and essential oil (EO) fractions of *C. citratus* on the pCMUT plasmid. **(A)** Representative agarose gel electrophoresis showing the supercoiled (SF) and relaxed (RF) forms of the plasmid for each treatment; **(B)** quantification of DNA breaks per kbp derived from the gels. C(−): plasmid DNA (negative control); C(+): plasmid DNA irradiated with UVC and treated with T4 endonuclease V (positive control); samples treated with 0.1, 0.5, 1.0, 2.0, and 4.0 mg/mL for 30 min at 37 °C. Error bars represent the SEM. (*) *p* < 0.05, Dunnett’s test versus C(−).

To evaluate the effect of extended exposure, the maximum non-genotoxic concentration (4.0 mg/mL) was tested at increasing incubation times (0.5, 1, 2, and 4 h) (Figure 2).

**FIGURE 2:**
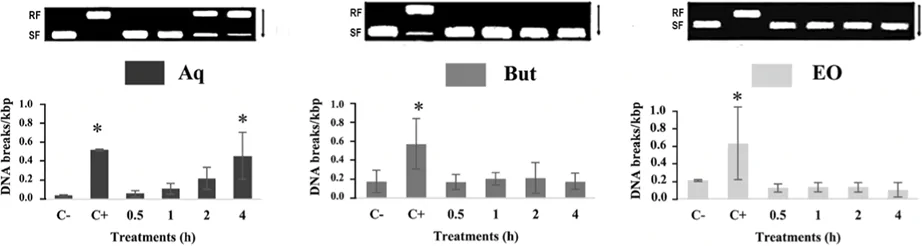
Effect of increasing incubation time of aqueous (Aq), butanolic (But), and essential oil (EO) fractions of *C. citratus* on the pCMUT plasmid. C(−): Plasmid DNA; C(+): irradiated DNA (UVC) and T4 endonuclease V; DNA treated with the concentration 4.0 mg/mL for 0.5, 1, 2 and 4 h respectively at 37 °C. (*) p < 0.05 Dunnett’s test when compared to C (−).

The results showed that the aqueous fraction caused a significant increase in DNA strand breaks after 4 h of exposure (*p* = 0.032), whereas no significant change was observed at 0.5, 1, or 2 h (p = 0.819, 0.624, and 0.487, respectively). In contrast, the butanolic and essential oil fractions did not cause any detectable changes in this parameter at any time point.

### 3.2 Assessment of oxidative damage

For the evaluation of oxidative damage, a concentration of 4.0 mg/mL of each fraction was selected, corresponding to the highest level that did not show genotoxic effects after 30 min of exposure in the previous assays. This decision allowed the establishment of a sensitive experimental point that maximized the probability of detecting oxidative lesions without the confounding effects of direct strand breaks. Thus, the choice of 4.0 mg/mL was based on methodological safety and toxicological relevance, ensuring that the observed effects could be specifically attributed to the pro-oxidative mechanisms of the compounds present in the fractions rather than nonspecific degradation of plasmid DNA.

The assessment of FPG enzyme inhibition revealed that none of the studied chemical fractions inhibited the enzyme’s activity (Figure 3A). Following this result, the oxidative damage evaluation was conducted (Figure 3B).

**FIGURE 3:**
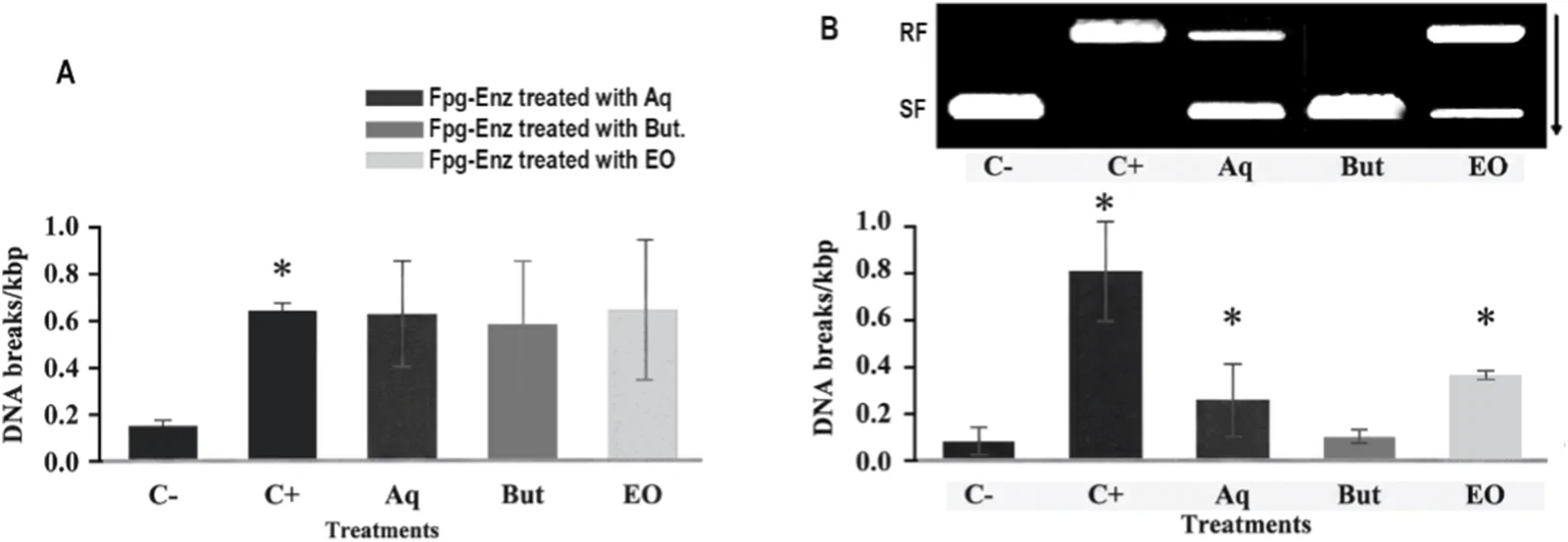
Evaluation of the effect of chemical fractions of *C. citratus* on FPG enzyme **(A)** and oxidative capacity of chemical fractions on naked DNA **(B)**. C(−): Plasmid DNA irradiated DNA (UVA), C(+): irradiated DNA (UVA) and FPG enzyme; Aq: aqueous fraction; But: butanolic fraction; EO: essential oil fraction. (*) p < 0.05 Dunnett’s test when compared to C(+) and C(−), respectively.

Figure 3A, shows that preincubation of the FPG enzyme with any of the three fractions tested at 4 mg/mL does not affect its capacity to recognize and excise UVA-oxidized DNA (500 kJ/m^2^), when compared to C(+), a mandatory premise to the oxidative evaluation. The results presented in Figure 3B clearly indicate that the aqueous and essential oil fractions of *C. citratus* induce significant oxidative DNA damage, evidenced by the marked increase in the frequency of plasmid DNA strand breaks compared to the negative control (C-). In contrast, the butanolic fraction did not induce the formation of the RF DNA band under the analyzed conditions.

### 3.3 Chemical analysis of the aqueous and butanolic fractions by HPLC

The chromatographic profile of different fractions obtained from the aqueous extract is presented in Figure 4. Peaks were identified based on their elution, UV-Vis absorption features, experimental molecular formula, and MS/MS fragmentation pattern (Costa et al., 2016; Figueirinha et al., 2008).

**FIGURE 4:**
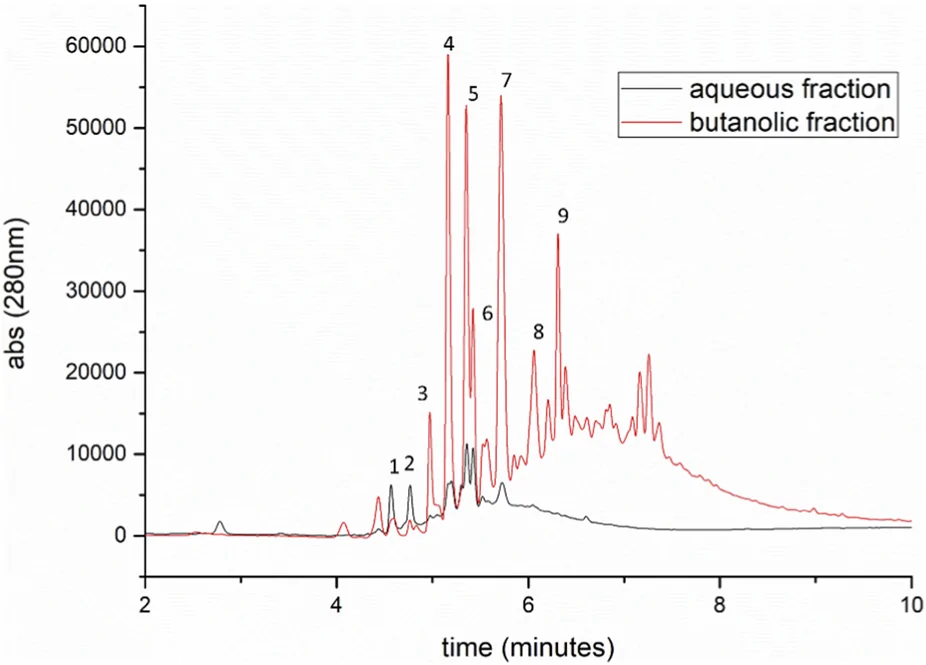
Chromatographic profile of the aqueous (black) and butanolic (red) fractions extracted at 280 nm. 1–9: Natural products identified in the aqueous and butanolic fractions.

High-performance liquid chromatography coupled with mass spectrometry (HPLC-MS) enabled the identification of a broad range of phenolic compounds present in both the aqueous and butanolic fractions of *C. citratus*. Both extracts contained polyphenols, particularly hydroxycinnamic acid derivatives (λmax 310–328 nm) and flavonoids (λmax 330–348 nm), including chlorogenic acid isomers (peaks 1 and 2, most abundant in aqueous fraction), carlinoside (4), isoschaftoside (5), 6-C-pentosyl-8-C-pentosyl luteolin (6), and 7-O-neohesperosyl luteolin (7, most abundant in butanolic fraction). These compounds are known for their antioxidant and biological activities. Although qualitatively similar, the butanolic fraction exhibited a richer overall profile and contained three additional compounds not found in the aqueous fraction: caffeoylglycerol (peak 3), cassiaoccidentalin B (8), and helonioside A (9). These qualitative and quantitative differences in phytochemical composition may underlie the distinct genotoxic and oxidative behaviors observed in the experimental assays.

It is important to note that the essential oil fraction was not subjected to phytochemical characterization in this study. Consequently, the interpretation of its biological activity relies on previously published compositional profiles of *C. citratus* essential oils.

## 4 Discussion

The results obtained in this study confirm that the chemical fractions of the aqueous extract of *C. citratus* (DC) Stapf do not induce DNA strand breaks after 30 min of exposure to increasing concentrations, which is consistent with previous studies using plasmid as an *ex vivo* DNA model (González-Pumariega et al., 2016). Statistical analysis confirmed the absence of significant differences compared to the negative control (p > 0.05), supporting the non-genotoxic nature of the fractions under acute exposure conditions in this *ex vivo* model. This experimental consistency with previous studies confirms the reproducibility of the *ex vivo* model for detecting primary DNA damage induced by natural extracts.

However, when the incubation time was extended, the aqueous fraction exhibited genotoxic behavior, with a progressive increase in strand breaks that reached statistical significance after 4 h of exposure. This finding represents a novel contribution by demonstrating that the aqueous fraction, like the total extract (González-Pumariega et al., 2016), although harmless in the short term, can induce primary DNA damage under prolonged exposure conditions, likely related to the compounds in the aqueous fraction. This experimental evidence also allows us to reinterpret data previously obtained in bacterial models, in which cytotoxicity and activation of the SOS response were reported in *E. coli* when treated (2h) with the aqueous fraction (Fuentes-León et al., 2017). While these results could be due to indirect cellular processes, the current data confirm a direct interaction between the fraction and naked DNA, complementing previously reported effects.

Furthermore, FPG was employed to reveal oxidative lesions (Gilboa et al., 2002), that do not directly produce DNA strand breaks, thereby enabling a more sensitive and specific evaluation of the pro-oxidant potential of the *C. citratus* fractions. This study represents the first report showing that the aqueous and essential oil fractions induce oxidative damage to plasmid DNA, while the butanol fraction does not exhibit such activity. It is worth noting that, although the butanolic fraction has demonstrated cytotoxicity in bacteria (Fuentes-León et al., 2017), its effects do not appear to be mediated by genotoxic mechanisms, suggesting non-genetic cellular targets.

Although the aqueous and butanolic fractions contain some compounds in common, the difference in genotoxic behavior could be explained by variations in the proportion and polarity of the compounds. From a phytochemical perspective (Table 1), the chromatograms obtained for the aqueous and butanolic fractions show distinguishable profiles, qualitatively and quantitatively.

**TABLE 1:** Compounds and experimental data found in the chemical fractions (aqueous and butanolic) of *C. citratus* (DC) Stapf.

Peak	t_R_ (min)	Compound	Fraction		Molecular formula	λ_max_ (nm)	MS^1^ [M−H]^−^ (*m/z*)	MS^2^ fragments (*m/z*)	Peak area (a.u.)		Rel. area (%)	
			Aq	But					Aq	But	Aq	But
1	4.57	Chlorogenic acid (isomer)	✓	✓	C_16_H_18_O_9_	326	353.09	191, 173	272.9	158.3	21.4	1.6
2	4.76	Chlorogenic acid (isomer)	✓	✓	C_16_H_18_O_9_	326	353.09	191, 173	270.4	101.1	21.2	1.0
3	4.97	Caffeoylglycerol	–	✓	C_12_H_14_O_6_	325	253.07	179, 161, 135	*n.d.*	610.0	*n.d.*	6.1
4	5.16	Carlinoside	✓	✓	C_26_H_28_O_15_	310	579.13	519, 489, 459, 399	252.3	2678.9	19.8	26.9
5	5.35	Isoschaftoside	✓	✓	C_26_H_28_O_14_	330, 272	563.14	545, 503, 473, 443, 383, 353	256.6	1621.0	20.2	16.2
6	5.57	6-*C*-Pentosyl-8-*C*-pentosyl luteolin	✓	✓	C_25_H_26_O_14_	346, 272	549.12	459, 489, 399, 531	27.8	445.6	2.2	4.5
7	5.71	7-*O*-Neohesperosyl luteolin	✓	✓	C_27_H_30_O_15_	347	593.15	447, 285	193.2	3147.3	15.2	31.5
8	6.21	Cassiaoccidentalin B	–	✓	C_27_H_28_O_14_	346	575.14	531, 473, 429, 411, 367	*n.d.*	264.5	*n.d.*	2.7
9	6.31	Helonioside A	–	✓	C_32_H_38_O_17_	328	693.20	517, 499, 337	*n.d.*	948.9	*n.d.*	9.5
Total identified	​	​	​	​	​	​	​	​	1273.2	9975.6	100.0	100.0

Compounds such as two chlorogenic acid isomers, carlinoside, isoschaftoside, and two glycosylated luteolin derivatives were identified in both fractions. Peaks were also detected exclusively in the butanol fraction, such as peak 3 (caffeoylglycerol), peak 8 (cassiaoccidentalin B), and peak 9 (helonioside A) (Costa et al., 2016; Figueirinha et al., 2008; Sousa et al., 2021). This qualitative distinction is noteworthy, as it implies the presence of unique compounds with differential bioactive potential, which refutes the perception of similarity in the phytochemical composition of both fractions.

From previous research, it is known that carlinoside, isoschaftoside, and luteolin derivatives are C-glycosylated flavonoids (Xiao et al., 2016); with non-genotoxic evidence in the revised scientific literature. For example, luteolin, present in both fractions, is a compound with a broad bioactive spectrum. Its recognized anticancer and antioxidant activity has been attributed to the modulation of signaling pathways without evidence of direct DNA damage mechanisms (Imran et al., 2019; Rauf et al., 2024). Given its presence in both the genotoxic (aqueous) and non-genotoxic (butanolic) fractions, it is reasonable to assume that the cytotoxicity observed in previous studies is due to mechanisms independent of DNA damage.

According to reports in the literature, there is no evidence of the genotoxic potential of caffeoylglycerol. It is an ester of caffeic acid and glycerol, and it acts as a potent free radical scavenger (Meng et al., 2018). Similarly, there are no published studies directly assessing the genotoxicity of helonioside A, an homoisoflavonoid glycoside structurally related to isoflavones, with cytotoxic activity in Hela cells (Panda et al., 2012). Cassiaoccidentalin B, belongs to the C-glycosylated luteolin family with cytotoxicity capacity in macrophages, an option as anti-inflammatory (Francisco et al., 2014), hence no evidence of genotoxicity was found.

Particular attention is drawn to the presence of peaks 1 and 2 in both fractions, corresponding to isomeric forms of chlorogenic acid. There is previous evidence that the genotoxic effect of this compound is very low or ambivalent (dose-dependent). At normal physiological concentrations obtained from the diet, it is predominantly antigenotoxic and antioxidant. However, *in vitro*, at very high concentrations, it exhibits pro-oxidant effects through reactive oxygen species (ROS), in several models (Burgos-Morón et al., 2012; Hou et al., 2017; Kai et al., 2021; Rakshit et al., 2010), which can cause DNA damage, such as single-strand breaks (SSBs) and the formation of 8-oxo-2′-deoxyguanosine.

From a quantitative point of view, the differences between the common peaks (1, 2, 4, 5, 6, and 7) are significant, especially those corresponding to the isomers of chlorogenic acid. The higher relative concentration of these two compounds in the aqueous fraction could also promote pro-oxidant effects *ex vivo*, as chlorogenic acid undergoes auto-oxidation to generate H_2_O_2_ and ROS, leading to oxidative DNA damage, as previously reported in comet assay studies (Burgos-Morón et al., 2012). This mechanism may be attenuated in the butanolic fraction due to lower concentrations and a less polar matrix that limits ROS reactivity.

Regarding the essential oil fraction, its inclusion in the study is justified for toxicological and ethnobotanical reasons. Although a chromatogram of its composition is not presented in this work, the essential oil fraction is part of the set of fractions generated during the traditional decoction process, which involves simultaneous aqueous extraction and the release of volatiles. From an experimental and toxicological perspective, assessing its genotoxic potential allows for the identification of risks associated with volatile compounds with alternative exposure routes, such as inhalation or indirect ingestion. However, it is recognized that analyzing the results of this fraction based on the phytochemical composition derived from the literature limits the ability to directly associate the observed effects with specific compounds. This lack of direct chemical characterization of the essential oil fraction (e.g., by GC-MS) represents a limitation of the present study, as it prevents the identification of the specific constituent, or combination of constituents, responsible for the observed biological effects.

The oxidative effects observed in the essential oil fraction, in the absence of DNA strand breaks, suggest an alternative mechanism of interaction with DNA, which could explain the cytotoxic effects previously reported by our group in bacterial models exposed to this same fraction (Fuentes-León et al., 2017). Numerous studies have demonstrated that *C. citratus* essential oil exerts potent broad-spectrum antimicrobial activity and, at the same time, its cytotoxicity has been studied in several cancer cell lines (Bayala et al., 2018; Hacke et al., 2022; Maksimović et al., 2025). In the literature, these effects have been mainly attributed to the predominant monoterpenes in *C. citratus* essential oil: citral (geranial + neral), β-myrcene (Luang-In et al., 2024), geraniol (Hacke et al., 2022), and limonene—also found in Cuban varieties (Pinto et al., 2015). However, because the essential oil fraction used in the present study was not characterized by GC-MS or an equivalent analytical method, the presence, relative abundance, and individual contribution of these compounds to the observed oxidative and DNA-damaging effects cannot be confirmed from the current data; their involvement is therefore inferred from previous literature rather than demonstrated in the tested fraction. Interestingly, essential oils have been shown to exhibit dose-dependent cytotoxic and pro-oxidant effects (Ishaya et al., 2025; Maksimović et al., 2025). Therefore, the evidence obtained here supports and reinforces the previously reported antimicrobial and cytotoxic profile of *C. citratus* essential oil, pointing to a common underlying mechanism driven by oxidative stress and DNA-related damage.

Finally, this genotoxic data appears to contradict previous reports on the DNA-protective effects of *C. citratus,* but this discrepancy disappears upon closer examination of the doses and exposure times. The results reveal a biphasic mode of action: the total extract induces DNA strand breaks at concentrations equal to or greater than 2 mg/mL, but only after 90 min of exposure (González-Pumariega et al., 2016), and shows no genotoxicity in the *E. coli* SOS-chromotest (Vernhes et al., 2016). In both papers, photoprotection is evident at lower doses. In the same mode, chemical fraction evaluation shows cytotoxicity and genotoxicity following butanol > aqueous and oils, similarly at high doses (Fuentes-León et al., 2017), whereas lower doses were associated with photoprotective effects (Fuentes-León et al., 2017). The results here obtained reinforce this idea, since the oxidative damage generated beyond DNA breaks under long-term exposure aligns with a U-shaped dose-response profile, where low doses are antigenotoxic and high doses become pro-oxidant, genotoxic, and cytotoxic. It should be emphasized that this U-shaped profile is not inferred from the single concentration assayed in the present time-course experiment, but emerges from the integration of these results with the protective effects previously reported at lower doses for the same plant material (González-Pumariega et al., 2016; Vernhes et al., 2016; Fuentes-León et al., 2017). These findings underscore the need for comprehensive toxicological assessments of *C. citratus*-derived fractions that consider the duration of exposure, the physicochemical properties of each fraction, and the context of traditional use, to support a robust, evidence-based safety assessment for the development of *C. citratus*-based products.

## 5 Conclusion

Taken together, this work provides *ex vivo* evidence of genotoxicity and oxidative damage induced by specific fractions of *C. citratus*, which helps explain and expand previous findings in bacterial models. The aqueous fraction emerges as the one with the greatest potential risk due to its high content of pro-oxidant phenolic compounds, while the butanol fraction shows a safer chemical profile under the conditions evaluated. The essential oil fraction, although with limited interpretive scope, exhibits pro-oxidant behavior. These findings reinforce the need for thorough phytochemical and toxicological evaluations before promoting the pharmacological or cosmetic use of plant fractions based on partial evidence.

## Data Availability

The original contributions presented in the study are publicly available. This data can be found here: Zenodo https://doi.org/10.5281/zenodo.22976240.
